# The Lack of Interest in Hygiene

**Published:** 1884-01

**Authors:** 


### The Lack of Interest in Hygiene.

"People hate advice," said a prominent publisher recently, when talking about a book on the prevention of disease, "when they are well, they never think about their health; when they are sick, they go to the doctor." That this is true, all admit; that this is deplorable, those of us who know the value of hygiene, sadly deplore.

Can we not rouse in the people an appreciation of the value of prevention, and cause them to realize how much they have their own physical welfare in their own hands? If we can accomplish this desirable purpose, it can only be done by united effort--by the united effort of the medical profession, who must constantly preach the doctrine of prevention.

The constant dropping of water will wear away the hardest rock, and so the constant preaching of hygiene, and this only, will ultimately wear away the rock of popular indifference.

The following sentence from Dr. Gairdner's address before the Sanitary Congress at Glasgow well illustrates the tardiness with which the people accept the tenets of hygiene:

"That it should have taken two centuries to reduce to a practical rule in the navy of Great Britain the measure by which Commodore Lancaster saved his crew from scurvy in 1603, is one of those facts which can not be got over, and which show that, in a practical sense, preventive medicine and the effective study of the causes of disease is a thing of yesterday as compared with the ages of effort, not wholly unsuccessful, devoted to the cure of diseases.

